# Metadata of agricultural long-term experiments in Europe exclusive of Germany

**DOI:** 10.1016/j.dib.2021.107322

**Published:** 2021-08-25

**Authors:** Meike Grosse, Marlen C. Ahlborn, Wilfried Hierold

**Affiliations:** Leibniz Centre for Agricultural Landscape Research (ZALF), Germany

**Keywords:** Agricultural experiment, Crop rotation experiment, Fertilization experiment, Long-term effects of agriculture, Mineral fertilization, Organic fertilization, Soil organic carbon, Tillage experiment

## Abstract

Agricultural long-term experiments (LTEs) are an important research infrastructure for agriculture, plant and soil sciences. The aim of this metadata compilation is to make LTEs easier to find and to facilitate networking. LTEs are here defined as agricultural experiments with a minimum duration of 20 years and research in the context of soil and yield. An extensive literature review was conducted to identify LTEs in Europe exclusive of Germany, because Germany's LTEs were published before. Sources were scientific papers as well as other articles, books, trial guides and websites. The following information was searched for and compiled in this dataset, if available: site and name of the LTE, start and end (if appropriate), holding institution, type of land use (e.g. field crops or grassland), research theme, website (if available), participation in networks, measured parameters, farming category (i.e. conventional or organic), size of the LTE area, longitude and latitude of the LTE, experimental setup including factors, treatments, randomization and replication, number of plots, size of the plots, crop rotation, soil type, substrate, texture, literature which was written in the context of the LTE data, and AGROVOC keywords. LTE from the following countries are included: Austria, Belarus, Belgium, Bulgaria, Czech Republic, Denmark, Estonia, Finland, France, Great Britain, Hungary, Italy, Moldova, Norway, Poland, Romania, Serbia, Slovenia, Spain, Sweden, Switzerland, Ukraine. In total, 186 LTEs could be identified. The LTEs were classified according to the following research themes: fertilization, tillage, crop rotation, other. The majority of LTEs have the research theme “fertilization” (*n* = 125). Thirty LTEs have the research theme “crop rotation”, 26 LTEs have the research theme “tillage”, and 26 LTEs have “other” research themes. The following networks could be identified: GLTEN (Global long-term experiment network), ILTER (International long-term ecological research), IOSDV (International Organic Nitrogen Fertilization Experiment), NLFT (National Long-term Fertilization Trials, Hungary), RetiBio 2 (Italy). The oldest LTE was set up 1843, but the largest number of LTEs was established in the second half of the 20th century. Most of the LTEs are held by a scientific institution, i.e. 88 LTEs are held by a non-university scientific institution and 81 LTEs are held by a university or university of applied sciences. The link to the holding institution is provided whenever possible to facilitate contacting.

## Specifications Table

**Table:** 

Subject	Agricultural Sciences
Specific subject area	Agricultural long-term experiments are an important basis for soil and agricultural sciences. A compilation of metadata from LTEs in Europe shall simplify findability and networking.
Type of data	Figures Tables
How data were acquired	An extensive literature review was conducted to identify LTEs. The search terms were “long-term field experiment”, “long-term experiment”, “long-term field trial” and “long-term trial”, as well as the German terms “Dauerfeldversuch”, “Dauerdüngungsversuch”, “Dauerversuch”, “Langzeitfeldversuch” and “Langzeitversuch”. AGROVOC (https://agrovoc.fao.org/browse/agrovoc/en/) keywords were added for each LTE that best describe the respective LTE.
Data format	Primary (raw) and secondary data
Parameters for data collection	Site, name, start, end (if appropriate), duration, holding institution, type of land use (e.g. field crops or grassland), research theme, website, participation in networks, measured parameters, farming category (i.e. conventional or organic), size of the LTE area, longitude, latitude, experimental setup, soil type, substrate, texture, literature which was written in the context of the LTE data, and AGROVOC keywords.
Description of data collection	The information was collected out of scientific papers as well as other articles, books, trial guides and websites. The duration was calculated: end year (if appropriate) or 2021 minus start year. Longitude and latitude were searched in google maps if not available in literature.
Data source location	See https://metadata.bonares.de/smartEditor/rest/upload/3af1450b-6000-4f38-a6e9-89d9c7b726bd/SourcesLTEEurope.pdf
Data accessibility	Repository name: BonaRes Repository Data identification number: 10.20387/bonares-eff3-0mb4 Direct URL to data: https://doi.org/10.20387/bonares-eff3-0mb4 Instructions for accessing these data: open access

## Value of the Data

• The data provide an overview of LTEs in Europe and exceed previous compilations in terms of quantity and depth of information.
• The dataset makes it easier to find suitable LTEs or institutions for cooperation.
• Although it is not a complete overview, this data set allows conclusions to be drawn about which topics are being researched with LTEs in Europe and which topics may be missing.

## 1 Data Description

LTEs are here defined as agricultural experiments with a minimum duration of 20 years and research in the context of soil and yield. LTEs which will reach the 20-year threshold by 2024 (= end of project funding phase) are also included. After a map with metadata for LTEs in Germany [1] and the complete metadata set have been published [2], information on LTEs in other European countries is now to be published. In total, 186 LTEs could be identified. The following information was compiled in this dataset: site and name of the LTE, start and end (if appropriate), holding institution, type of land use (e.g. field crops or grassland), research theme, website of the LTE or of the holding institution, participation in networks, measured parameters, farming category (i.e. conventional or organic), size of the LTE area, longitude and latitude of the LTE, experimental setup including factors, treatments, randomization and replication, number of plots, size of the plots, crop rotation, soil type, substrate, texture, and literature which was written in the context of the LTE data. Table 1 gives an overview of the number of LTE for which the respective information is available.

**Table 1:** Type of information compiled in the dataset and number of LTE for which the respective information is available (sorted according to frequency).

Type of information	Number LTE
Country	186
Name of the site	186
Research theme	185
LTE name	182
Start year	182
Type of land use	182
Holding institution	173
End year	172
Website	142
Measured parameters	117
Texture	109
Experimental setup	106
Soil (sub) type	99
Exact position	90
Size plots	87
Size of LTE	82
Crop rotation	78
Number plots	70
Farming category	59
Factor 1	54
Replication	54
Member of Networks	49
Substrate	46
Factor 3	42
Factor 2	39
Literature	24
Randomization	21

Table 2 indicates the number of LTEs per country which is recorded in the data set. The type of land use is specified. The LTEs are classified as fertilization LTE, tillage LTE, or crop rotation LTE, if one factor is fertilization, tillage, or crop rotation (multiple nominations possible). The majority of LTEs have the research theme “fertilization” (n = 125). Thirty LTEs have the research theme “crop rotation”, 26 LTEs have the research theme “tillage”, and 26 LTEs have “other” research themes (Table 2).

**Table 2:** List of countries for which LTEs are known. Summary of the total number of LTEs per country and type of land use. The number of LTEs in which research on fertilization, tillage, crop rotation or other topics is conducted is mentioned (multiple nominations possible).

Country	Land use	Number LTE	Fertilization LTE*	Tillage LTE*	Crop rotation LTE*	Other LTE*
Austria	Field crops	6	5	2	2	
	Grassland	10	9			1
Belarus	Field crops	2	1			
Belgium	Field crops	4	3	1	1	
Bulgaria	Field crops	1	1			1
	unknown	4	4			
Czech Republic	Field crops	10	10		2	
Denmark	Energy crops	1				1
	Field crops	9	8	2	1	
Estonia	Field crops	2	2			
Finland	Field crops	4		2		2
	Grassland	1				1
France	Field crops	8	7	3		1
Great Britain	Clover	1	1			1
	Energy crops	1				1
	Field crops	22	18	1	4	8
	Field crops/Grassland	2			1	2
	Grassland	1	1			
Hungary	Field crops	14	14	1	1	
Italy	Field crops	5	1	2		3
	Pomiculture	1	1			
	Vegetables	2				2
Moldova	Field crops	1				
Norway	Field crops	10	4	5	1	
Poland	Field crops	7	2	3	4	1
Romania	Field crops	13	12	1		
Serbia	Field crops	1	1		1	
Slovenia	Field crops	2	2		1	
Spain	Field crops	4	1	3	1	
Sweden	Field crops	34	14		10	
Switzerland	Field crops	1	1			1
	Grassland	1	1			
Ukraine	Field crops	1	1			

⁎ Multiple nominations possible

**Fig. 1:**
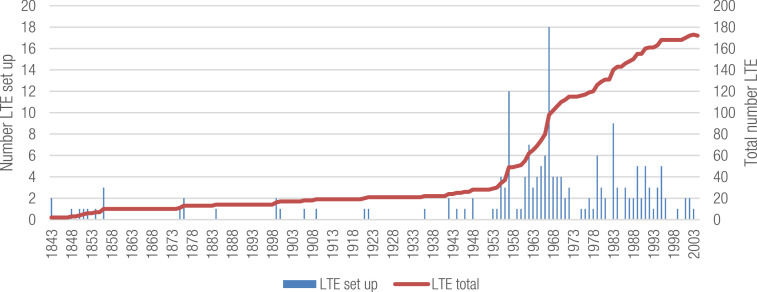
Number of LTE set up per year and total number of LTE.

The oldest LTE was set up 1843 (Fig. 1). The largest number of LTEs was established in the second half of the 20th century. Now sixteen LTEs are finished. The total number of LTEs shows the number of established LTEs minus the number of finished LTEs (Fig. 1).

The following five networks could be identified: GLTEN (Global long-term experiment network), ILTER (International long-term ecological research), IOSDV (International Organic Nitrogen Fertilization Experiments), NLFT (National Long-term Fertilization Trials, Hungary), and RetiBio 2 (Italy). Table 3 shows the number of LTEs belonging to each network.

**Table 3:** List of LTE Networks that could be identified and number of LTE belonging to each network.

Network	Number LTE
GLTEN (Global long-term experiment network)	25
NLFT (National Long-term Fertilization Trials, Hungary)	9
IOSDV (International Organic Nitrogen Fertilization Experiments)	8
RetiBio 2 (Italy)	5
ILTER (International long-term ecological research)	2

Eighty-eight LTEs are held by a non-university scientific institution (Table 4). Eighty-one LTEs are held by a university or a university of applied sciences. We could not identify the type of holding institution for 13 LTEs. For four LTEs the holding institution is another institution than a non-university scientific institution or a University/University of applied sciences (e.g. a state authority).

**Table 4:** List of holding institution categories.

Type of LTE holding institution	Number LTE
Non-university scientific institution	88
University / University of applied sciences	81
unknown	13
Other Institution	4

## 2 Experimental Design, Materials and Methods

An extensive literature review was conducted to identify LTEs in Europe. Sources were scientific papers as well as other articles, books, trial guides and websites. The search terms were “long-term field experiment”, “long-term experiment”, “long-term field trial” and “long-term trial”, as well as the German terms “Dauerfeldversuch”, “Dauerdüngungsversuch”, “Dauerversuch”, “Langzeitfeldversuch” and “Langzeitversuch”. Attention was given to LTEs with a minimum duration of 20 years in the context of soil research, i.e., the objects of research should at least include soil properties and yield as an important soil function. The setup of each trial should allow for statistical analyses, i.e., have clearly defined factors, replications and as much as possible a static design. Lysimeter experiments were excluded because they were considered as an own category. Some reasons for this exclusion are that soils are often transferred and not undisturbed in lysimeter experiments and tillage has to be conducted by hand instead of machines, which can bias some results.

LTEs were only included if more information than just site and start was available. Special emphasis was placed on the holding institution, whose website was linked whenever possible. Not all LTEs from other lists, e.g. in Bai et al. [3] or in Debreczeni and Körschens [4] are included in this dataset. If the duration was too short or if it was impossible to identify the holding institution or some more details, the LTE was excluded.

All information was put into an excel sheet.

We partially assigned the information to categories in order to sort the LTE more efficiently. This was the case for holding institution (categories: Non-university scientific institution, university / university of applied sciences, or other institution than that) and research theme (categories: crop rotation, fertilization, tillage, or other). Each LTE was classified in one or more of these categories if one or more factors could be assigned to these research themes. The class “other” entails all research themes that could not be grouped into the first three and appeared only in a few LTE cases, so that a separate group was not justified. Two or more factorial experiments were sorted in all relevant classes, i.e., multiple nominations were possible. The current duration in 2021 of each LTE was calculated from its start year (and possibly the end if it is a finished experiment). The coordinates were either taken from the literature, if they were given there, or searched via google maps [5]. AGROVOC [6] keywords were added for each LTE that best describe the respective LTE. In addition, there are added generalizing terms of AGROVOC that make it easier to find similar LTEs on specific topics.

## CRediT Author Statement

**Meike Grosse:** Conceptualization, Methodology, Data curation, Investigation, Visualization, Writing – Original draft preparation; **Marlen C. Ahlborn:** Data curation; **Wilfried Hierold**: Conceptualization, Methodology.

## Declaration of Competing Interest

The authors declare that they have no known competing financial interests or personal relationships which have or could be perceived to have influenced the work reported in this article.
